# LCN2 depletion aggravates sepsis-induced liver injury by regulating PTGS2-dependent ferroptosis

**DOI:** 10.7150/ijms.98246

**Published:** 2024-10-21

**Authors:** Yun Jiang, Zhi-Tian Jiang, Gang Zhao, Jing-Wen Cai, Jie Song, Jing Wang, Zhen Zhou, Qian Wang, Qi-Hua Ling

**Affiliations:** 1 Department of Hepatology, Shuguang Hospital, Shanghai University of Traditional Chinese Medicine, Shanghai, China.; 2 Department of Outpatient Office, Shuguang Hospital, Shanghai University of Traditional Chinese Medicine, Shanghai, China.; 3 Department of Emergency Internal Medicine, Shuguang Hospital, Shanghai University of Traditional Chinese Medicine, Shanghai, China.

**Keywords:** sepsis, liver injury, ferroptosis, LCN2, PTGS2

## Abstract

**Background:** Sepsis-induced liver injury (SILI) is an independent risk factor for organ dysfunction and mortality in critical care units.

**Methods:** In this study, the roles of lipocalin 2 (LCN2) in SILI were investigated because LCN2 expression was increased in liver tissues of the septic mice induced by caecal ligation and puncture (CLP), as well as in hepatocytes treated with lipopolysaccharide (LPS). To evaluate liver injury in mice, the levels of alanine transaminase (ALT), aspartate transaminase (AST), and alkaline phosphatase (ALP) were measured in both serum and liver tissues. Oxidative stress was evaluated by measuring the levels of malondialdehyde (MDA), superoxide dismutase (SOD), and glutathione (GSH) in serum and liver samples. Additionally, ferroptosis was assessed by examining the expression of prostaglandin endoperoxide synthase 2 (PTGS2), solute carrier family 7 member 11 (SLC7A11), and glutathione peroxidase 4 (GPX4) in liver tissue.

**Results:** The results demonstrated that LCN2 depletion significantly exacerbated SILI, oxidative stress, and ferroptosis. Moreover, in *in vitro* sepsis model, LCN2 overexpression notably ameliorated LPS-induced cell injury, oxidative stress, and ferroptosis by inhibiting PTGS2 expression.

**Conclusion:** In conclusion, our study provides evidence that LCN2 depletion aggravates SILI by regulating PTGS2-mediated ferroptosis.

## Introduction

Sepsis is widely recognized as the main cause of death in critical care units, and its survivors often face more severe pathology and higher readmission rates compared to those who have not experienced sepsis^1^, ^2^. Multiple organs, including the liver, brain, heart, and lung, can be affected by sepsis^2^-^4^. Notably, liver injury has emerged as an early prognostic indicator of poor outcomes in sepsis patients, and interventions aimed at restoring liver function have shown promise in improving prognosis^5^, ^6^. However, the precise mechanism of sepsis-induced liver injury (SILI) remains unclear.

Ferroptosis is a type of regulated cell death that has recently gained interest in scientific research, characterized by the buildup of reactive oxygen species (ROS) and free iron within cells^7^. It has been established that iron metabolism and oxidative stress-related processes contribute significantly to the development of ferroptosis^7^, ^8^. Key processes involved in ferroptosis include the accumulation of free iron, depletion of glutathione (GSH), and the buildup of lipid oxidative damage leading to cell membrane degeneration^8^, ^9^. Interruption of these processes has been shown to impede the development of ferroptosis. Several genes, such as solute carrier family 7 member 11 (SLC7A11) and glutathione peroxidase 4 (GPX4), act as negative regulators of ferroptosis by inhibiting ROS production^10^, ^11^. Conversely, positive regulators like prostaglandin endoperoxide synthase 2 (PTGS2) and transferrin receptor 1 (TFR1) promote ROS production, thereby enhancing ferroptosis^10^. Notably, ferroptosis has been associated with several disease-related cell death events, such as liver damage.

LCN2 is a member of the lipocalin family, a group of secreted proteins that functions as transporters for small lipophilic molecules, including iron and lipopolysaccharides, within the bloodstrdies^12^. Previous studies have demonstrated that lipocalin 2 (LCN2) is strongly activated in inflamed organs such as spleen, kidney, lungs, heart, and liver^13^. In a lipopolysaccharide (LPS)-induced sepsis model, LCN2 knockout exacerbates inflammation, oxidative stress, organ damage by regulating iron homeostasis^14^. However, the relationship between LCN2 and ferroptosis in SILI, as well as its potential regulatory mechanisms, remains unclear.

In this study, we explored the involvement of LCN2 in SILI in cecal ligation and puncture (CLP)-induced sepsis model. Our findings revealed a significant increase in LCN2 expression in both sepsis-induced liver tissues and hepatocytes treated with LPS. Interestingly, LCN2 depletion exacerbated SILI, oxidative stress, and ferroptosis. Further analysis revealed that LCN2 overexpression suppressed hepatocyte ferroptosis by inhibiting PTGS2 expression. These findings highlight the protective role of LCN2 in SILI by mitigating oxidative stress and hepatocyte ferroptosis. Collectively, our data offer valuable insights into the intricate mechanisms underlying SILI, and it suggests that LCN2 may be a promising target for the treatment of SILI.

## Materials and Methods

### Animal

Eight-week-old male C57 BL/6 mice were obtained from Caygen (Suzhou, China) and kept them in a controlled environment with a temperature of 24°C ± 2°C, 50% relative humidity, and a 12-hour light/dark cycle. The mice had free access to food and water. All procedures involving animals were performed in compliance with the guidelines set forth by the Experimental Animal Welfare and Ethics Committee of the Shanghai University of Traditional Chinese Medicine (No PZSHUTCM200320006) in accordance with ARRIVE guidelines.

### CLP model of sepsis

Mouse model of sepsis was established by carrying out a CLP, as previously described 15. In brief, the mice were anesthetized using sevoflurane and a 2-cm incision was made along the midline of the abdomen. The cecum was then ligated halfway along its distal end after moving the stool towards the tip. Bacterial peritonitis was induced by puncturing the cecum with a 22-gauge needle. At the end of the experiment, all the mice were euthanized by pentobarbital.

### LCN2 knockdown

Recombinant adenoviruses expressing shRNA targeting LCN2 (Ad-shLCN2) under the mouse albumin promoter were purchased from Genechem Co., LTD (Shanghai, China) and were used for LCN2 depletion *in vivo*, as previously described^16^, ^17^. The Ad-negative control (Ad-NC, Genechem) was used as the control. During this experiment, a solution containing viruses was prepared by diluting them in phosphate-buffered saline (PBS). Each mouse then received an injection of the viral solution into the tail vein at a concentration of 2 × 10^9^ plaque-forming units. On the 21st day following viral injection, the CLP procedure was performed. Liver and serum samples were collected 16 hours after CLP-induced sepsis for further analysis.

### Primary hepatocyte isolation and culture

Primary hepatocytes were isolated from 8-week-old C57 BL/6 mice according to previously established protocols^16^, ^18^. Briefly, hepatocytes were extracted by digestion with type II collagenase (Gibco™, 17101015), and subsequent centrifugation through a 25-50% Percoll gradient. The isolated primary hepatocytes were then grown in DMEM culture medium enriched with 10% FBS and 0.01 mM dexamethasone with 5% CO_2_ at 37°C.

### LCN2 overexpression in hepatocytes

Recombinant adenoviruses encoding the full-length LCN2 cDNA (Ad-LCN2) were purchased from Genechem Co., LTD. The Ad-vector (Genechem) was used as the control. Primary hepatocytes were treated with Ad-LCN2 according to the manufacturer's instructions and the LCN2 overexpression was validated by quantitative real-time PCR (qRT-PCR) and western blot assays.

### Cell septic model

The septic model was established in hepatocytes by LPS treatment as previously described^19^, ^20^. In brief, primary hepatocytes were stimulated with LPS (100 ng/mL) for 24 hours.

### Immunohistochemistry (IHC) and hematoxylin-eosin (H&E) staining

Mouse liver tissues were collected after perfusion with cold PBS and fixed with 10% formalin. Then the tissues were embedded in paraffin and cut into 4-µm-thick sections. For IHC staining, the liver tissue sections were treated with citrate buffer (pH 6.0) for 5 min at 108 °C and pretreated with 3% hydrogen peroxide (H2O2) for 15 min at room temperature. After being blocked with normal goat serum for 20 min, the sections were incubated with primary antibodies against LCN2 overnight at 4°C. Then the tissue sections were treated with a secondary antibody conjugated to horseradish peroxidase (HRP) and incubated at room temperature. DAB solution (100 μl) was added to the sections. After being restained with hematoxylin, the sections were visualized using an inverted microscope (Olympus, Tokyo, Japan). H&E staining was carried out to detect the morphological changes of mouse liver tissues.

### Western blot assay

Liver tissues and hepatocytes were lysed in RIPA assay buffer (Invitrogen, USA) supplemented with a protease inhibitor cocktail (Invitrogen, USA). Protein concentration was determined using a BCA kit (Invitrogen, USA). The proteins were separated using SDS-PAGE and transferred to PVDF membranes. After being blocked by 5% nonfat milk, PVDF membranes were incubated with primary antibodies against LCN2 (1:1000, ab125075, Abcam), PTGS2 (1:1000, ab179800, Abcam), GPX4 (1:1000, ab125066, Abcam), SLC7A11 (1:1000, ab175186, Abcam), and beta-actin (1:5000, ab8226, Abcam) overnight at 4°C. The membranes were then incubated with HRP-conjugated anti-mouse or anti-rabbit secondary antibody (1:5000) at room temperature. Finally, the blots were visualized by ECL reagents (P0018S, Beyotime, Shanghai, China).

### qRT-PCR

Total RNA was isolated from liver tissues and hepatocytes using Trizol reagent (Invitrogen, USA) and quantitated with Bioanalyzer 2100 (Agilent, CA, USA). Reverse transcription PCR was performed using the Super-SMART-PCR-cDNA-Synthesis Kits (TaKaRa, JPN). qPCR was conducted on an ABI-7500 Real-Time PCR System (Applied Biosystems, USA) using PowerUp SYBR Green Mix (Invitrogen, USA) following the protocol: 95°C for 10 minutes, 35 cycles of 95°C for 15 s, 59°C for 20 s, and 72 °C for 25 s, and 72 °C for 30 s. The fold changes of RNA transcripts were determined using the 2^-ΔΔCt^ method, with 18s as the reference gene.

### Enzyme-linked immunosorbent assay (ELISA)

The levels of ALT, AST, and ALP were assessed using a mouse ALT ELISA kit (ab282882, Abcam), mouse AST ELISA kit (ab263882, Abcam), and mouse ALP ELISA kit (ab285274, Abcam) according to the manufacture's protocol, respectively.

The levels of MDA, GSH, and SOD were assessed using a mouse MDA ELISA kit (MOFI01410, Biomol, Hamburg, Germany), mouse GSH ELISA kit (MOEB2568, Assay Designs, Ann Arbor, MI, USA), and mouse SOD ELISA kit (ab285309, Abcam) according to the manufacture's protocol, respectively. The content of Fe^2+^ was measured using an iron assay kit (ab83366, Abcam). The ROS level was measured using a mouse ROS ELISA kit (KTE71621, Wuhan, China). The levels of IL-6, IL-1β, and TNF-α were assessed by commercial kits (IL-6: BMS603-2, ThermoFisher, MA, USA; IL-1β: BMS6002-2TEN, ThermoFisher; TNF-α: BMS607-3FIVE, ThermoFisher), according to the manufacture's protocol.

### Statistics

All statistical analyses were performed using SPSS21 (IBM, NY, USA). Data were presented as mean ± standard deviation (SD) from three independent experiments. The significance between two groups was assessed using Student's t-test, and the significance between more than two groups was determined using one-way ANOVA followed by the Tukey-Kramer multiple comparisons test. A p-value less than 0.05 was considered statistically significant.

## Results

### LCN2 was increased in liver tissues of CLP model and in LPS-treated hepatocytes

**Figure 1:**
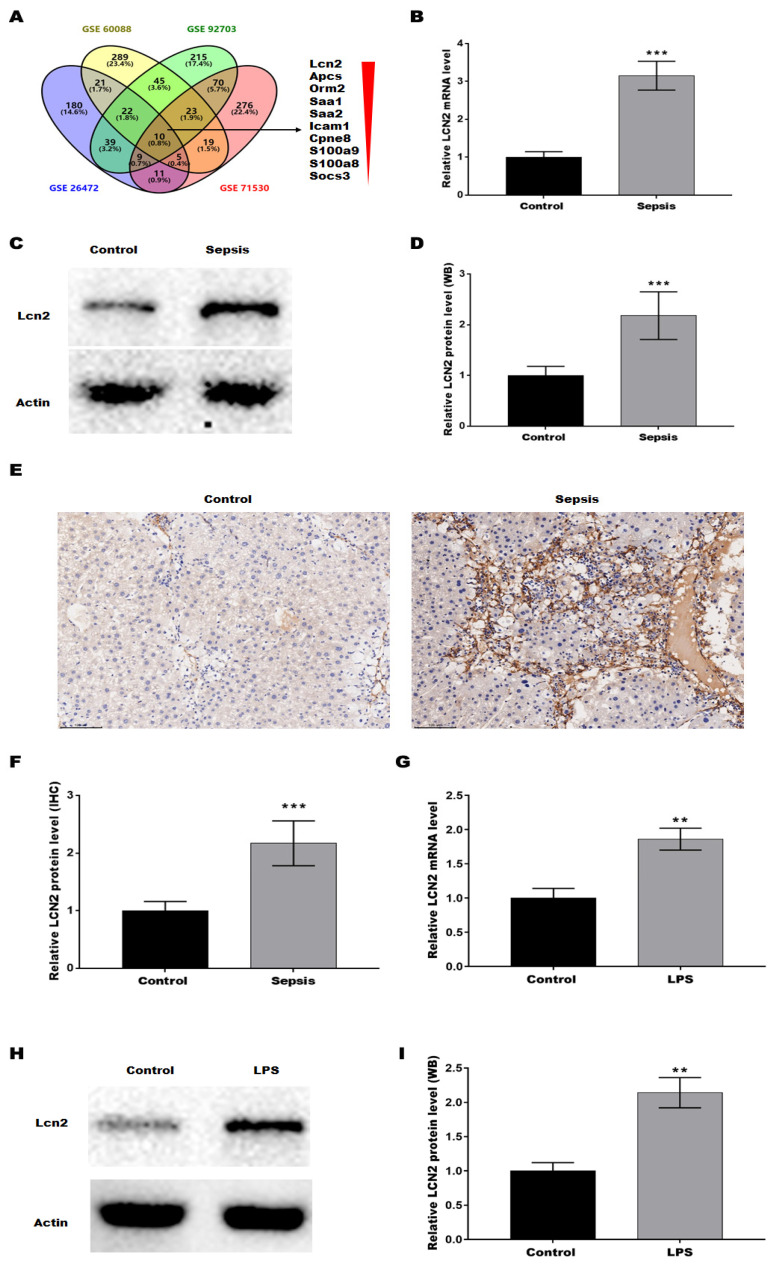
** LCN2 was upregulated in liver tissues of CLP model and in LPS-treated hepatocytes.** (A) The datasets (GSE6008, GSE92703, GSE26472, and GSE71530) were obtained from the GEO. The DEGs were screened out based on |Log FC| > 1 and p<0.05, and then the common DEGs across the 4 datasets were determined by Venn diagram analysis. (B) LCN2 mRNA levels were assessed by qRT-PCR in liver tissues of the control and septic mice. (C and D) Western blot and quantitative analysis of LCN2 protein levels in liver tissues of the control and septic mice. (E and F) IHC and quantitative analysis of LCN2 expression in liver tissues of the control and septic mice. (G) LCN2 mRNA levels were assessed by qRT-PCR in primary hepatocytes before and after LPS treatment. (H and I) Western blot and quantitative analysis of LCN2 proten levels in primary hepatocytes before and after LPS treatment. Data were presented as mean ± SD (n = 5 per group). **p < 0.01, ***p < 0.001.

To verify the key genes associated with the SILI, four datasets (GSE6008, GSE92703, GSE26472, and GSE71530) were obtained from the Gene Expression Omnibus (GEO, https://www.ncbi.nlm.nih.gov/gds/). The differentially expressed genes (DEGs) were screened out based on |Log FC| > 1 and p < 0.05, and then the common DEGs across the 4 datasets were determined by Venn diagram analysis, revealing a total of 10 common DEGs (Lcn2, Apcs, Orm2, Saa1, Saa2, Icam1, Cpne8, S100a9, S100a8, and Socs3) (Figure 1A). Given that LCN2 was the most significantly different gene and was reported to be associated with liver injury in alcoholic hepatitis^21^ and non-alcoholic steatohepatitis^22^, LCN2 was selected to investigate whether it is involved in SILI. Firstly, LCN2 expression was assessed in CLP-induced septic mice by qRT-PCR. Figure 1B showed that the LCN2 mRNA level was markedly increased in liver tissues of septic mice compared with control mice. Moreover, LCN2 protein level was analyzed by western blot and IHC. Consistent with the qRT-PCR results, LCN2 protein expression was significantly increased in liver tissues of septic mice compared with control mice (Figures 1C-F). In addition, LCN2 expression was also examined in the LPS-induced sepsis cell model. Figures 1G-I displayed that LCN2 was significantly increased in the LPS-induced sepsis cell model. These data suggest that aberrant LCN2 expression may be associated with SILI.

### LCN2 depletion promoted liver injury in CLP-induced sepsis model

**Figure 2:**
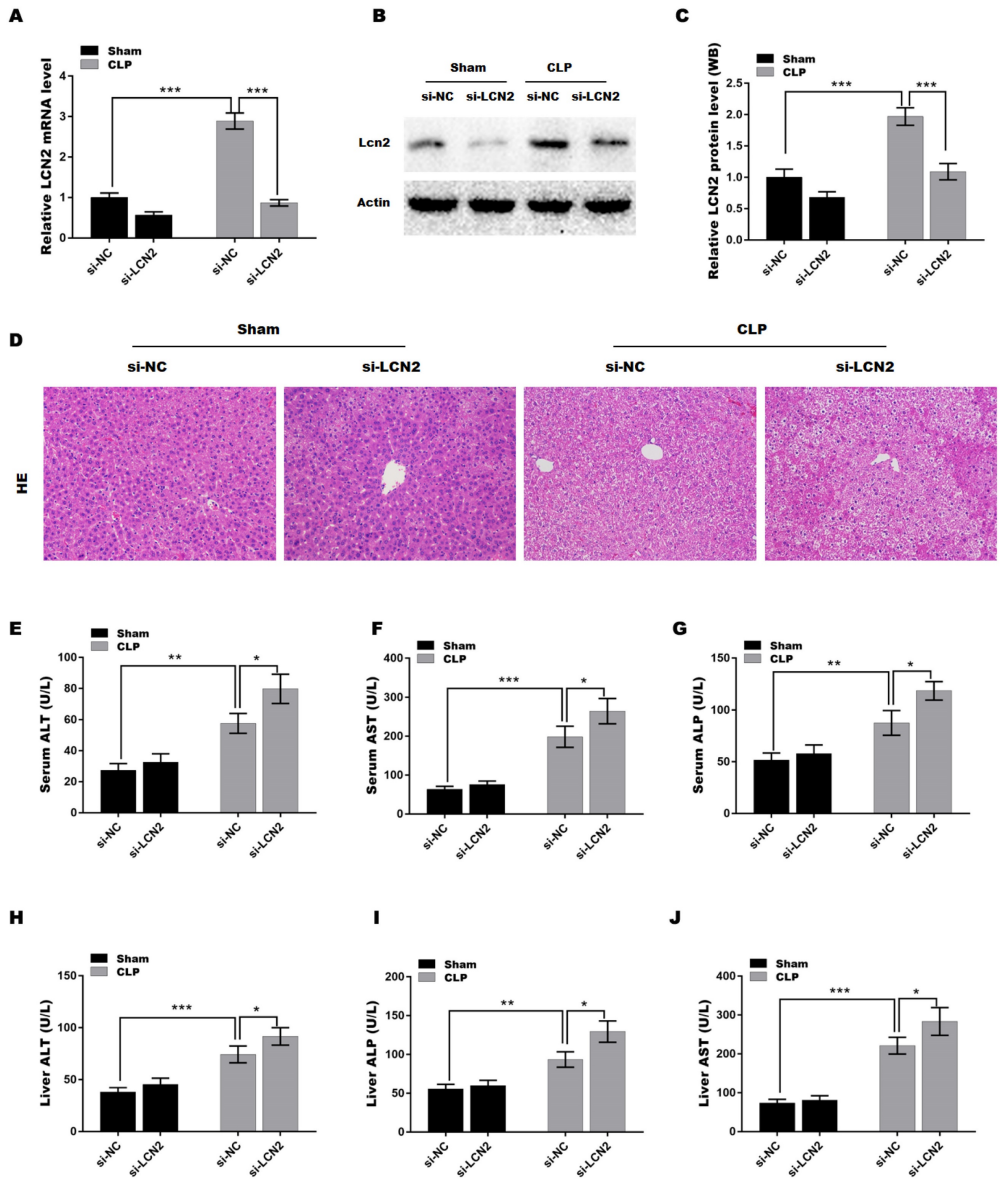
** LCN2 depletion promoted liver injury in CLP-induced sepsis model.** A septic mouse model was established by CLP and then LCN2 was knocked down by tail vein injection of Ad-shLCN2. The following experiments are carried out. (A) LCN2 mRNA levels were assessed by qRT-PCR in liver tissues of the control and CLP mice before and after LCN2 depletion. (B and C) Western blot and quantitative analysis of LCN2 protein expression in liver tissues of the control and CLP mice before and after LCN2 depletion. (D) H&E staining of liver tissues of the control and CLP mice before and after LCN2 depletion. (E-G) Serum ALT, AST, ALP levels were assessed by ELISA. (H-J) Liver ALT, AST, ALP levels were assessed by ELISA. Data were presented as mean ± SD (n = 5 per group). *p < 0.05, **p < 0.01, ***p < 0.001.

To explore the function of LCN2 in sepsis-induced liver injury, a septic mouse model was established by CLP and then LCN2 was knocked down by tail vein injection of Ad-shLCN2. Figures 2A-C showed that LCN2 expression was successfully silenced at the mRNA (Figure 2A) and protein (Figures 2B and C) levels in liver tissues after Ad-shLCN2 treatment. Subsequently, H&E staining of liver tissues was carried out to assess liver injury. As depicted in Figure 2D, liver tissues of the septic mouse exhibited disrupted hepatic local inflammation, indicating liver injury. Surprisingly, LCN2 depletion exacerbated the CLP-induced liver injury, as evidenced by more severe alterations in the hepatic lobules and cords. Furthermore, we evaluated the levels of liver-specific enzymes, such as AST, ALT, and ALP, in the blood and liver tissues to further confirm liver damage. The levels of AST, ALT, and ALP were significantly elevated in the serum and liver tissues of septic mice compared to the control group (Figures 2E-J). Notably, LCN2 depletion further increased the levels of AST, ALT, and ALP in the serum and liver tissues of septic mice, indicating worsened liver injury in the depletion of LCN2.

### LCN2 depletion deteriorated oxidative stress and inflammation in CLP-induced sepsis model

**Figure 3:**
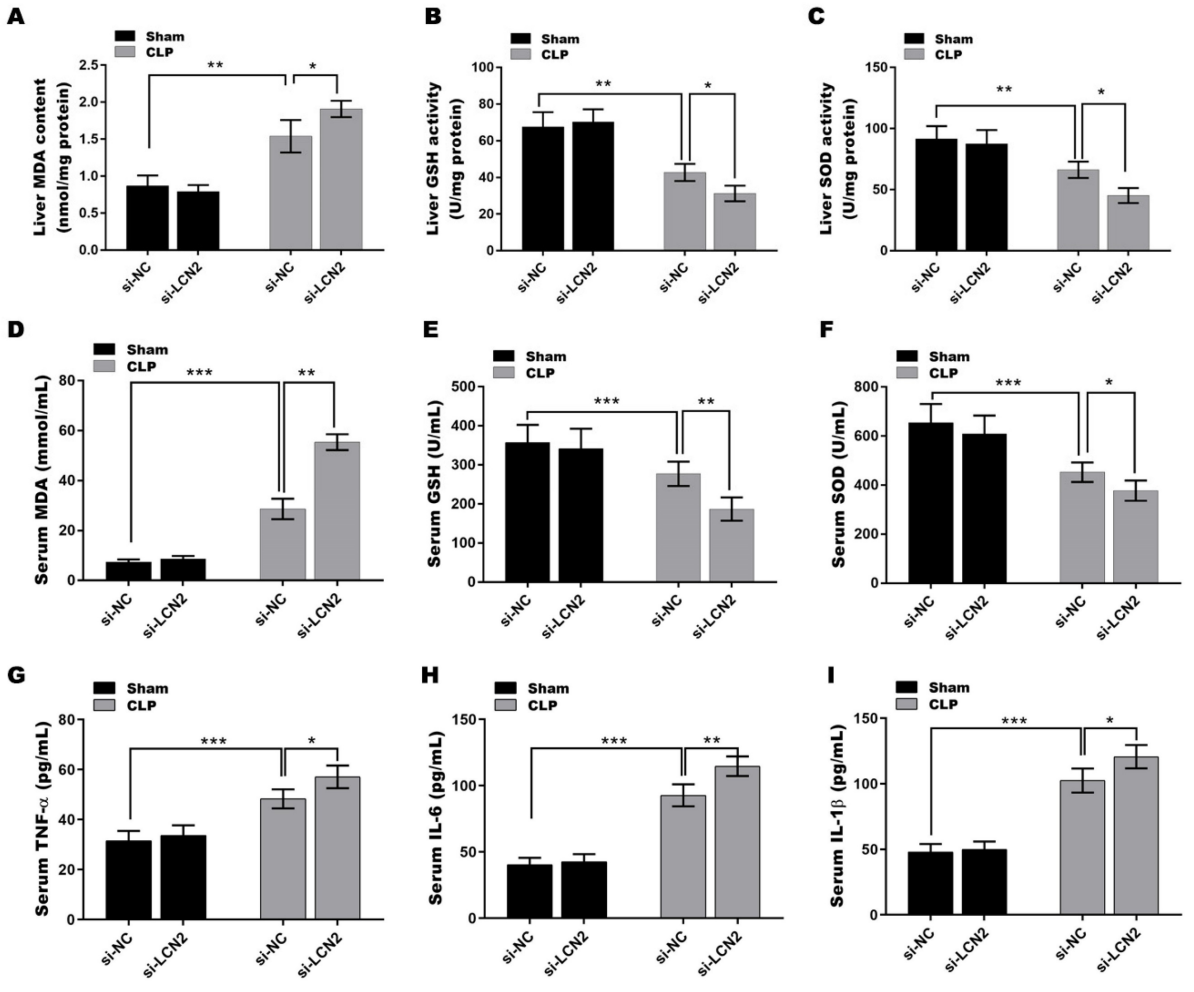
** LCN2 depletion deteriorated oxidative stress and inflammation in CLP-induced mice.** A septic mouse model was established by CLP and then LCN2 was knocked down by tail vein injection of Ad-shLCN2. The following experiments are carried out. (A-C) MDA, GSH, and SOD levels were assessed by ELISA in liver tissues of the control and CLP mice before and after LCN2 depletion. (D-F) MDA, GSH, and SOD levels were assessed by ELISA in serum of the control and CLP mice before and after LCN2 depletion. (G-I) The levels of TNF-α, IL-6, and IL-1βwas assessed by ELISA in serum of the control and CLP mice before and after LCN2 depletion. Data were presented as mean ± SD (n = 5 per group). *p < 0.05, **p < 0.01, ***p < 0.001.

It is well known that impaired antioxidant function and increased oxidative stress contribute to liver injury^23^, and LCN2 has been suggested to play an important role in modulating oxidative stress^24^. Based on this understanding, we next investigated whether LCN2 has a beneficial effect on septic liver injury through its antioxidant mechanisms. To this end, we examined the levels of key antioxidant defense markers, including SOD and GSH-Px, as well as the oxidative stress marker MDA, in the serum and liver tissues of septic mice after LCN2 depletion. As depicted in Figures 3A-F, a significant reduction in the activities of GSH and SOD was observed, coupled with a substantial increase in MDA levels, in liver tissues (Figures 3A-C) and serum (Figures 3D-F) of the septic mice compared to the control mice. LCN2 depletion exacerbated the CLP-induced oxidative stress in septic mice. Additionally, the levels of pro-inflammatory cytokines (TNF-α, IL-6, and IL-1β) were significantly increased in serum of the septic mice compared to the control mice, and LCN2 depletion further up-regulated the levels of these cytokines in septic mice (Figures 3G-I). These results demonstrate that LCN2 depletion exacerbated the CLP-induced oxidative stress and inflammation in septic mice.

### LCN2 depletion promoted CLP-induced ferroptosis in liver tissues

**Figure 4:**
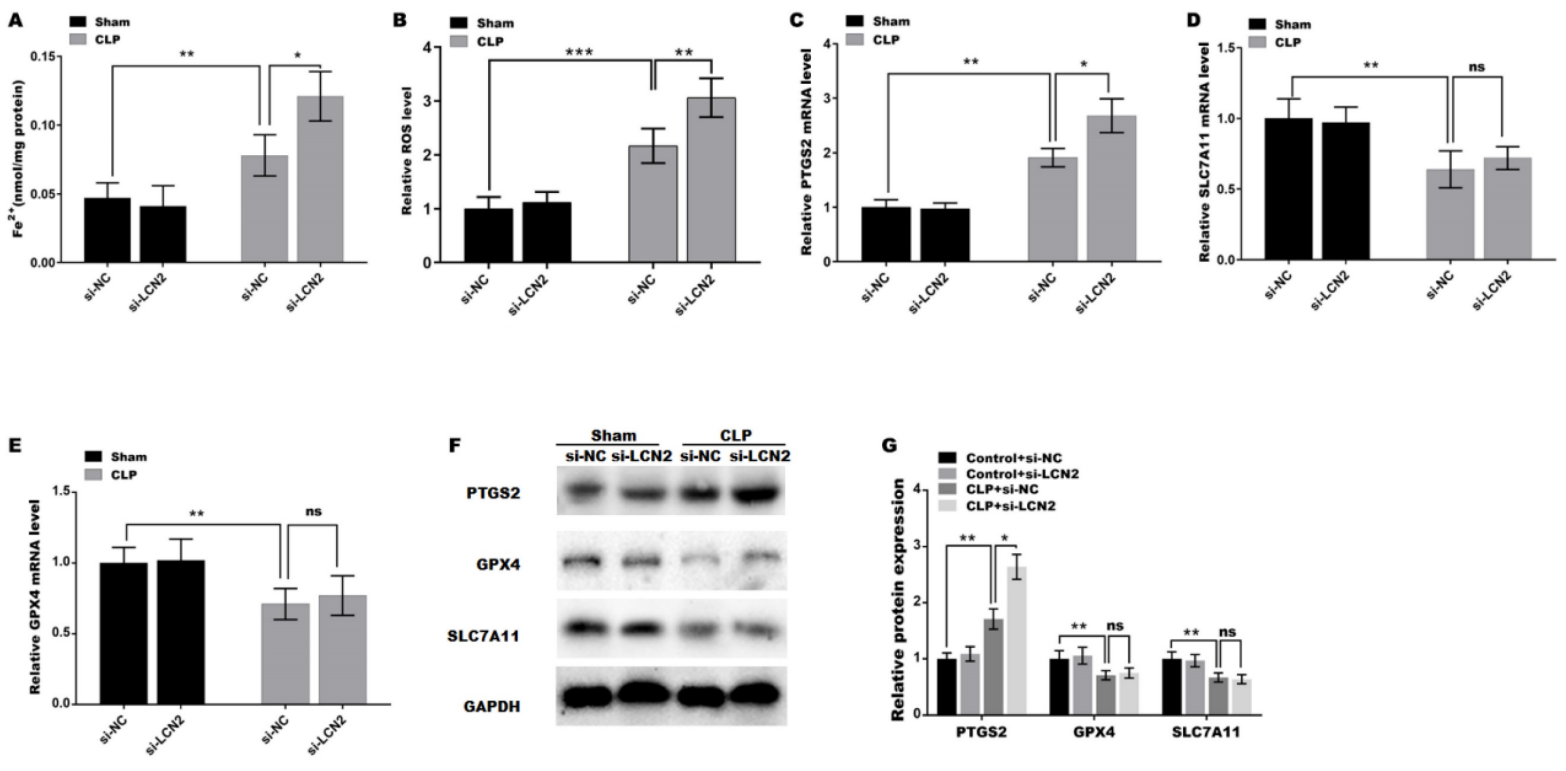
** LCN2 depletion promoted CLP-induced ferroptosis.** Fe^2+^ concentration (A) and ROS levels (B) were assessed by ELISA in liver tissues of the control and CLP mice before and after LCN2 depletion. (C-E) PTGS2, SLC7A11, and GPX4 mRNA levels were assessed by qRT-PCR in liver tissues of the control and CLP mice before and after LCN2 depletion. (F and G) Western blot and quantitative analysis of PTGS2, SLC7A11, and GPX4 protein levels in liver tissues of the control and CLP mice before and after LCN2 depletion. Data were presented as mean ± SD (n = 5 per group). *p < 0.05, **p < 0.01, ***p < 0.001.

Oxidative stress is a well-known contributor to the development of ferroptosis, and LCN2 has been implicated in the promotion of ferroptosis in various diseases^25^. Therefore, the roles of LCN2 in CLP-induced ferroptosis were next investigated. Figure 4A and B showed that the iron concentration and ROS level in liver tissues of the septic mice were significantly increased compared with control. Moreover, LCN2 depletion further elevated the iron content and ROS level in the liver tissues of septic mice (Figure 4A and B). These results are consistent with the trends of changes in MDA (Figure 3A), GSH (Figure 3B), and SOD (Figure 3C) mentioned earlier, indicating that CLP treatment caused ferroptosis in liver tissues, and LCN2 depletion further exacerbated ferroptosis, as previously described criteria for identifying ferroptosis^26^-^28^. To gain further insights into the molecular mechanisms involved, we assessed the expression of ferroptosis-related genes, namely PTGS2, GPX4, and SLC7A11, in liver tissues of the septic mice after LCN2 depletion by qRT-PCR (Figures 4C-E) and western blot assays (Figures 4F and G). The results demonstrated a marked decrease in the expression of GPX4 and SLC7A11, while PTGS2 expression was significantly increased in liver tissues of the septic mice, suggesting that sepsis induces ferroptosis in liver tissues. Notably, LCN2 depletion exacerbated ferroptosis by increasing PTGS2 expression, but not GPX4 or SLC7A11 (Figures 4C-G).

### LCN2 overexpression suppressed LPS-induced oxidative stress and ferroptosis by inhibiting PTGS2 expression in hepatocytes

**Figure 5:**
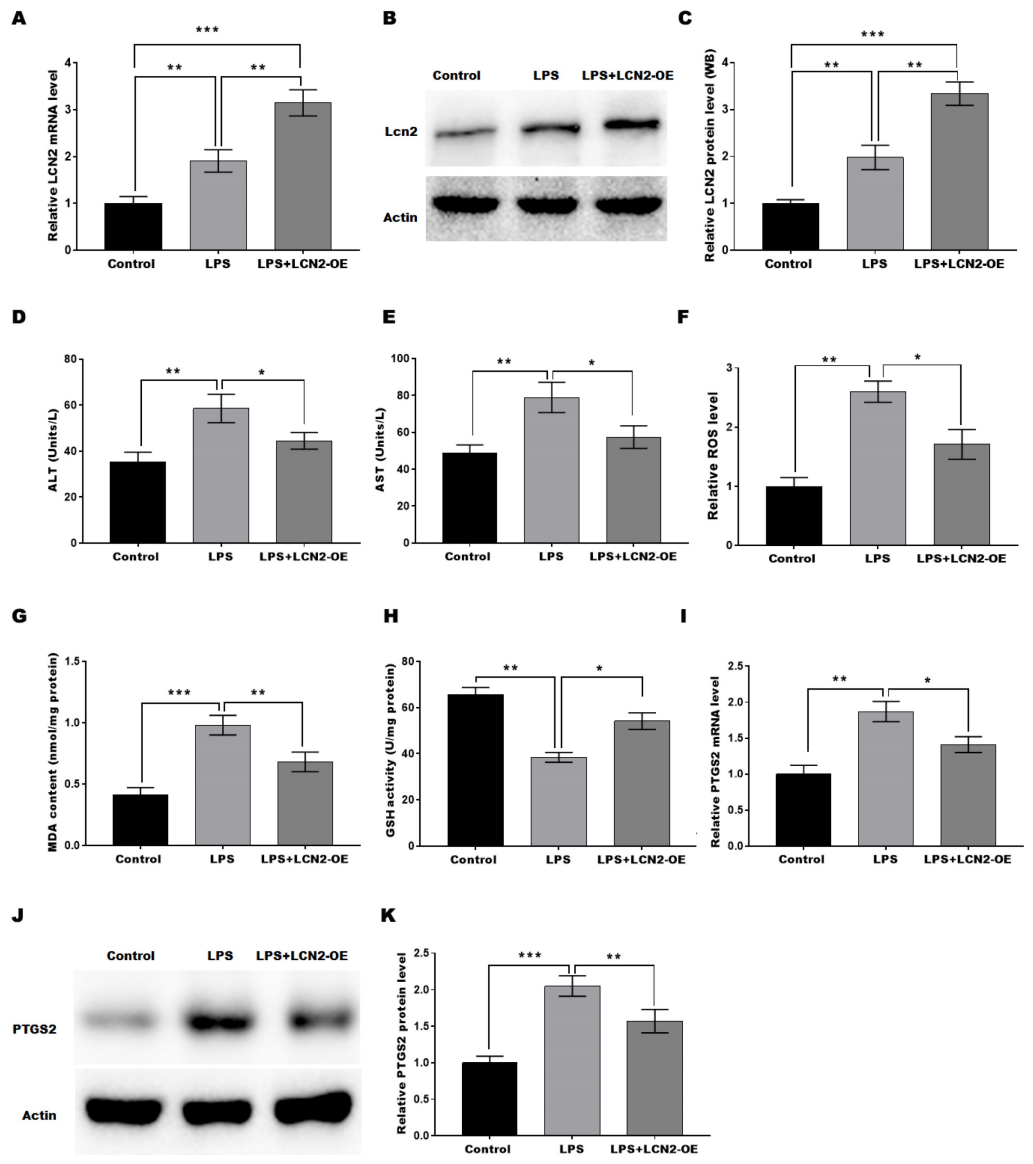
** LCN2 overexpression suppressed LPS-induced oxidative stress and ferroptosis of hepatocytes by inhibiting PTGS2 expression.** An *in vitro* sepsis model was established by stimulating hepatocytes with LPS and then LCN2 was overexpressed in the cell model. The following experiments are carried out. (A) LCN2 mRNA levels were assessed by qRT-PCR in control hepatocytes and LPS-treated hepatocytes before and after LCN2 overexpression. (B and C) Western blot and quantitative analysis of LCN2 protein levels in control hepatocytes and LPS-treated hepatocytes before and after LCN2 overexpression. ALT (D) and AST (E) levels were assessed by ELISA in supernatant of the control hepatocytes and LPS-treated hepatocytes before and after LCN2 overexpression. (F) ROS levels were measured by ELISA in the control hepatocytes and LPS-treated hepatocytes before and after LCN2 overexpression. (G and H) MDA and GSH levels were assessed by ELISA in control hepatocytes and LPS-treated hepatocytes before and after LCN2 overexpression. (I) PTGS2 mRNA (I) and protein (J and K) levels in control hepatocytes and LPS-treated hepatocytes before and after LCN2 overexpression were assessed by qRT-PCR and western blot assays, respectively. Data were presented as mean ± SD. *p < 0.05, **p < 0.01, ***p < 0.001.

To investigate the protective function of LCN2 in SILI, an *in vitro* sepsis model was established by stimulating hepatocytes with LPS, and then LCN2 was overexpressed in the cell model. LCN2 expression was markedly increased in Ad-LCN2-treated hepatocytes as validated by qRT-PCR (Figure 5A) and western blot (Figures 5B and C) assays. To assess the protective role of LCN2 in cell injury, the levels of AST and ALT in the medium of hepatocytes were measured. Figures 5D and 5E demonstrated that LPS stimulation markedly increased the levels of AST and ALT, suggesting hepatocyte damage. However, when LCN2 was overexpressed, the effect of LPS on ALT and AST production from hepatocytes was restored, suggesting that LCN2 overexpression inhibited the LPS-induced hepatocyte injury. Furthermore, we investigated whether LCN2 plays a protective role in SILI by regulating oxidative stress and ferroptosis. Figures 5F-H revealed that LPS stimulation resulted in a significant increase in ROS levels and MDA content, indicating oxidative stress in hepatocytes. However, when LCN2 was overexpressed, these effects were attenuated, with decreased ROS levels and MDA content, and increased GSH activity. These findings suggest that LCN2 plays a role in suppressing oxidative stress in hepatocytes during SILI. Moreover, Figures 5I-K demonstrated that LPS stimulation increased the expression of PTGS2, a marker of ferroptosis, in hepatocytes. However, LCN2 overexpression blocked this increase in PTGS2 expression. These results suggest that forced expression of LCN2 alleviates LPS-induced oxidative stress and ferroptosis in *in vitro* sepsis model.

## Discussion

Sepsis greatly affects the prognosis of patients with infectious diseases, and elucidating its mechanism is an important issue. In the current study, we demonstrated that LCN2 attenuates SILI by alleviating oxidative stress, inflammation, and PTGS2-dependent ferroptosis, indicating the potential role of interfering with the LCN2/oxidative stress/ferroptosis axis in the treatment of SILI.

LCN2 expression has been confirmed to be upregulated during bacterial infection^13^. Chan *et al.* reported that LCN2 is a crucial component of mucosal immune defense against pulmonary infection with *K. pneumonia*^29^. Hepatocytes secrete extracellular LCN2, which serves as a protective mechanism against systemic bacterial infection. Meanwhile, neutrophils transport LCN2 to the site of infection, where it contributes to local defense against bacterial infection through the formation of neutrophil extracellular traps^30^. These studies support the notion that LCN2 plays a crucial role in mucosal immune defense against bacterial infections and limiting systemic bacterial infections. Notably, a series of studies have demonstrated that LCN2 possesses a hepatoprotective function in liver damage. For instance, LCN2-deficient mice exhibited more severe liver damage when exposed to short-term applications of LPS, carbon tetrachloride, and Concanavalin A, further supporting the hepatoprotective function of LCN2^31^. LCN2 also exhibits a protective effect on the liver in the context of a fructose diet, as it coincided with reduced markers of oxidative stress and mitochondrial dysfunction^32^. In line with these studies, we found that LCN2 was upregulated in liver tissues of the sepsis-induced mice and in the LPS-treated hepatocytes. LCN2 depletion exacerbated the liver injury in CLP-induced septic mice, suggesting a protective function of LCN2 in SILI. However, the precise mechanisms underlying the protective effects of LCN2 are currently not fully understood.

In recent years, LCN2 has been implicated in various diseases through its regulation of ferroptosis, a kind of iron-dependent non-apoptotic cell death characterized by the accumulation of toxic lipid ROS. This process has gained attention in liver dysfunction, as the accumulation of ROS and excessive iron contribute to severe liver illnesses. Ferroptosis appears to play a dual role in the occurrence and development of liver injury. For instance, fibroblast growth factor 21 (FGF21) attenuates iron overload-induced liver injury and fibrosis by inhibiting ferroptosis^33^. Zhang *et al.* reported that embryonic lethal vision-like protein 1 (ELAVL1) regulates hepatic stellate cell ferroptosis involved in the development of liver fibrosis^34^, suggesting that ferroptosis could promote the development of liver fibrosis. Conversely, some studies have proposed that ferroptosis as a novel target to inhibit the activation of hepatic stellate cells (HSCs) and alleviate the fibrosis of the liver. Sui *et al.* found that magnesium isoglycyrrhizinate treatment obviously induces HSC ferroptosis by accelerating the accumulation of lipid peroxides and iron, whereas inhibition of ferroptosis by the specific inhibitor ferrostatin-1 (Fer-1) completely abolished the anti-fibrosis function of MgIG^35^. In our study, LCN2 depletion significantly increased oxidative stress in liver tissues of the CLP-induced septic mice, suggesting that LCN2 may improve liver injury by inhibiting ferroptosis.

The effects of LCN2 on oxidative stress and inflammation are complex and multifaceted in different types of diseases. While some studies indicate that LCN2 promotes oxidative stress and pro-inflammatory response, others suggest that LCN2 can inhibit oxidative stress and inflammation. For example, LCN2 treatment accelerates inflammation and oxidative stress by increasing iron accumulation in macrophages in acute lung injury (ALI)^36^. LCN2 knockdown decreases LPS-triggered oxidative stress and inflammation by suppressing mitogen-activated protein kinases (MAPK)/extracellular signal-regulated kinase (ERK) signaling in acute respiratory distress syndrome (ARDS)^24^. These studies indicate that LCN2 represents a promising therapeutic target for ALI and ARDS. On the other hand, LCN2 exhibits opposite effects on regulating oxidative stress and inflammation in other types of diseases. In lung adenocarcinoma, LCN2 silencing leads to cancer cell apoptosis by inactivating the nuclear factor E2-related factor 2 (Nrf2) and thus producing reactive oxygen species (ROS)^37^. In diabetic nephropathy, LCN2 depletion aggravates disease progression by increasing oxidative stress and inflammation through activating small mothers against decapentaplegic (Smad2/3) signaling^38^. LCN2 can also decrease iron-related ROS generation by increasing CD44 variant in ovarian clear cell cancer^39^. A recent study has identified the roles of LCN2 in inhibiting oxidative stress, alleviating organ injury, and decreasing mortality by regulating iron homeostasis in LPS-induced sepsis^14^. We further investigated the effects of LCN2 on SILI and its potential mechanisms based on the following two reasons: i) Ferroptosis is characterized by dysregulated iron homeostasis and the resulting increase in Fe^2+^, ROS, and MDA, and the decrease in GSH, SOD, and GPX4^40^. Given the roles of LCN2 depletion in increasing the levels of Fe^2+^ and ROS^14^, it is necessary to further investigate whether LCN2 silencing facilitates ferroptosis and reveal the potential mechanisms involved. ii) The CLP-induced sepsis model is different from the LPS-induced sepsis model^41^. LPS exposure triggers a rapid and potent inflammatory response, accompanied by early oxidative stress in organs. On the other hand, the response to CLP is less intense but tends to a longer duration^41^. Therefore, it is meaningful to verify the function of LCN2 in different sepsis models. In the present study, we reveal the protective role of LCN2 in SILI in CLP model of sepsis by attenuating PTGS2-dependent ferroptosis. Further investigation into the potential mechanisms by which LCN2 regulates oxidative stress and PTGS2 expression is essential.
